# Phytochemical profile, nutritional composition of pomegranate peel and peel extract as a potential source of nutraceutical: A comprehensive review

**DOI:** 10.1002/fsn3.3777

**Published:** 2024-01-08

**Authors:** Faiza Azmat, Mahpara Safdar, Hajra Ahmad, Muhammad Rizwan Jamil Khan, Junaid Abid, Muhammad Sadiq Naseer, Saurabh Aggarwal, Ali Imran, Urma Khalid, Syeda Mahvish Zahra, Fakhar Islam, Sadia Arif Cheema, Umber Shehzadi, Rehman Ali, Abdela Befa Kinki, Yuosra Amer Ali, Hafiz Ansar Rasul Suleria

**Affiliations:** ^1^ Department of Nutritional Sciences and Environmental Design Allama Iqbal Open University Islamabad Pakistan; ^2^ Department of Botany University of Okara Okara Punjab Pakistan; ^3^ Department of Food Science and Technology University of Haripur Haripur Pakistan; ^4^ Department of Clinical Nutrition NUR International University Lahore Pakistan; ^5^ Department of Mechanical Engineering Uttaranchal Institute of Technology Uttaranchal University Dehradun India; ^6^ Department of Food Sciences Government College University Faisalabad Pakistan; ^7^ Institute of Food Science and Nutrition University of Sargodha Sargodha Pakistan; ^8^ Food Science and Nutrition Ethiopian Institute of Agricultural Research Addis Ababa Ethiopia; ^9^ Department of Food Sciences, College of Agriculture and Forestry University of Mosul Mosul Iraq; ^10^ Department of Food Science, Faculty of Science University of Melbourne Melbourne Victoria Australia

**Keywords:** food applications, health benefits, nutraceuticals, nutritional composition, phytochemical profile

## Abstract

The current study focuses on *Punica granatum L.* (pomegranate) peel and peel extract and their use as functional foods, food additives, or physiologically active constituents in nutraceutical formulations. The pomegranate peel extract is a good source of bioactive substances needed for the biological activity of the fruit, including phenolic acids, minerals, flavonoids (anthocyanins), and hydrolyzable tannins (gallic acid). The macromolecules found in pomegranate peel and peel extract have been recommended as substitutes for synthetic nutraceuticals, food additives, and chemo‐preventive agents because of their well‐known ethno‐medical significance and chemical properties. Moreover, considering the promises for both their health‐promoting activities and chemical properties, the dietary and nutraceutical significance of pomegranate peel and pomegranate peel extract appears to be underestimated. The present review article details their nutritional composition, phytochemical profile, food applications, nutraceutical action, and health benefits.

## Graphical abstract

*Punica granatum L.* (pomegranate) peel and peel extract, their use as functional foods, food additives, or physiologically active constituents in nutraceutical formulations. The macromolecules found in pomegranate peel and peel extract have been recommended as substitutes for synthetic nutraceuticals, food additives, and chemo‐preventive agents because of their well‐known ethno‐medical significance and chemical properties.

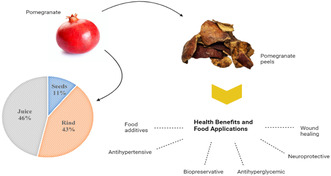

## 1 INTRODUCTION

Fruits and vegetables are the horticulture products that are consumed the most and produce waste items like peels and seeds. When fruits and vegetables are processed, a significant number of by‐products are produced, amounting to between 25% and 30% of the total amount of raw materials (Sagar et al., 2018). Skin, rind, seeds, and pomace make up the majority of the trash and are excellent sources of dietary fiber, polyphenols, and other potentially significant bioactive compounds including carotenoids. These phytochemicals can be employed in a wide range of industries, including the food business for the creation of enriched or functional foods, the pharmaceutical and health industries, as well as the textile industry. The food industry is one of the largest industries in the world majorly contributing towards economy. In any case, the rapid growth of the global population and the demands on the food supply chain will lead to a rapid increase in food production during the next 50 years. Approximately one‐third of the food produced for human consumption is lost or wasted worldwide (Despoudi et al., 2021). Food waste is caused by a combination of social and environmental factors. In addition to food wastage, the loss and waste of fruits and vegetables also has a negative impact on the resources such as land, water, fertilizers, and human labor, being lost in an indirect manner, inturn producing the majority of environmental waste (Andishmand et al., 2023; Sagar et al., 2018). A significant amount of fruits' and vegetables' waste can be recycled in a number of ways, such as immediate disposal in a waste repository, and processing using biotechnology to create artificial chemical rind. The pomegranate (*Punica granatum*), basically native to the Middle East, is now grown in the Mediterranean, China, India, South Africa, and the United States. Peel, seed, and juice are the three components that make up a pomegranate fruit (Kharchoufi et al., 2018). Pomegranate fruit contains a sizable amount of phenolic substances. Flavonoids, tannins, and phenolics with antimicrobial properties are all present in the fruit's peel, which accounts for 50% of the fruit's weight. These substances have the ability to remove free radicals and prevent the oxidation of lipids in fatty foods (George et al., 2019). The peel of the pomegranate contains a variety of bioactive substances, including quercetin, catechin, antioxidants, minerals, and vitamins, as well as several potential health benefits (Pirzadeh et al., 2020). These substances are essential for preserving health and provide defense against a number of crippling conditions like cardiovascular diseases (CVD, diabetes, and specific types of cancer (Gull et al., 2023). The numerous uses of pomegranate peel in the fields of food biopreservation, the development of nutraceutical products, and the production of value‐added items are also explored through current research. Since pomegranate peels naturally contain bioactive components, there is a great chance to use them as functional ingredients, maximizing the potential of by‐product resources and enhancing the overall value of the pomegranate. This review aims to present the composition and benefits that can be obtained by processing pomegranate by‐products, that is, its peel, to ultimately improve the health of individuals.

### 1.1 Nutritional composition

Pomegranate byproduct is enriched with numerous bioactive compounds, minerals, fibers, carbohydrates, fats, and proteins. A study was carried out at the Department of Post‐Harvest Technology, Bagalkot, Karnataka, India, to analyze the chemical and nutritional composition of peel. The study conducted pretreatment of the peel with a 2% salt solution for 10 minutes, which was then subjected to washing and drying. After pretreatment, dried peels were placed in a tray and were heated to 65°C for about 10 hrs. The dried peel was then mashed using a food processor and sieved through a 0.5 mm sieve. The hot air oven method was used to determine moisture content which was reported to be 7.27%. The ash content was found to be 4.32%. The differential method, Kjeldahl method, and Socs plus‐SCS‐6AS instrument were used to determine carbohydrate (66.51%), protein (3.74%), and total fat (0.85%) content, respectively. Crude fiber analysis was done using the Fibra plus‐FES‐6 instrument and it came out to be 17.31%. In addition to that, the peel powder also contained minerals such as calcium (342 mg), magnesium, potassium (148.64 mg), sodium (64.63 mg), phosphorus (118.3 mg), iron (6.35 mg), zinc (0.93 mg), manganese (0.78 mg), and copper (0.64 mg) per 100 g of dried pomegranate peel powder, which was determined by wet digestion. Total phenolic content (18.75 mg/g) was determined by the Folin Ciocalteu reagent (FCR) method (Ranjitha et al., 2018). Pomegranate peel is high in crude fiber, which has numerous health benefits and phenolic content due to which it has antioxidant, antimicrobial, antihypertensive, antilipidemic, and antidiabetic properties (Ain et al., 2023; Ranjitha et al., 2018) (Table 1).

**TABLE 1:** Chemical and nutritional composition of pomegranate peel powder.

Nutrients	Dried pomegranate peel powder	
Moisture (%)	7.27	
Ash (%)	4.32	
Carbohydrate (%)	66.51	
Protein (%)	3.74	
Total fat (%)	0.85	
Crude fiber (%)	17.31	
Total phenolic content (mg/g)	18.75	Ranjitha et al. (2018)
Calcium (mg/100 g)	342	
Magnesium (mg/100 g)	148.64	
Sodium (mg/100 g)	64.63	
Phosphorous (mg/100 g)	118.3	
Iron (mg/100 g)	6.35	
Zinc (mg/100 g)	0.93	
Manganese (mg/100 g)	0.78	
Copper (mg/100 g)	0.64	

### 1.2 Extraction of phytochemicals

**FIGURE 1:**
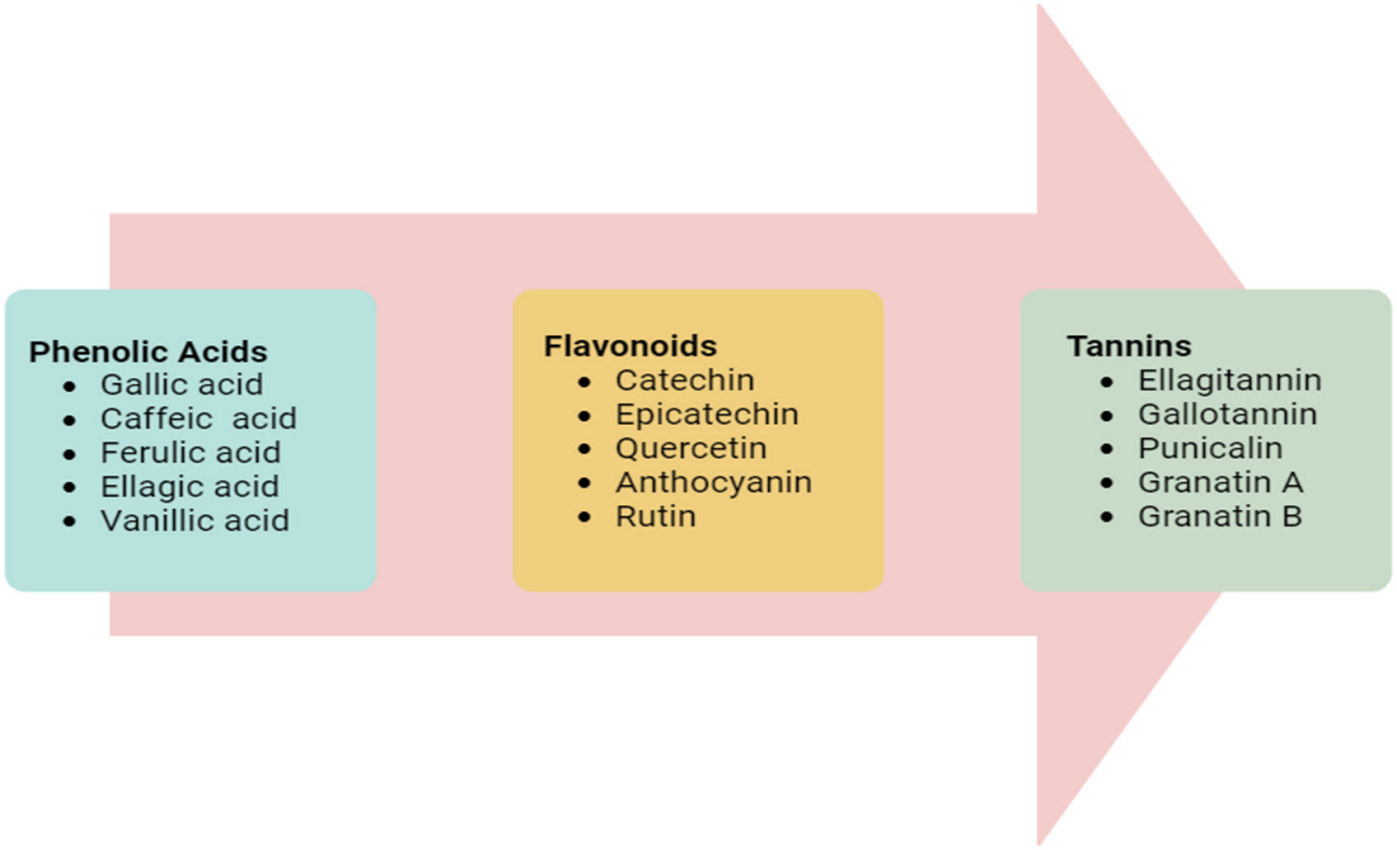
Bioactive components present in pomegranate peel.

A significant quantity of fruits is wasted in the processing industries, which pollutes the environment. This is true, especially for pomegranates. Although PoP is often thought of as waste, it contains a variety of polyphenolic compounds, including tannins, anthocyanins, and phenolic acids. Pomegranate peel includes considerable amounts of phenolic chemicals that are required for the fruit's biological activity, such as minerals and flavonoids (anthocyanins and catechins), hydrolyzable tannins (punicalagin, punicalin, ellagic acid, and gallic acid), and tannins. Phenols, tannins, anthocyanins, terpenoids, cycloglycosides, alkaloids, flavonoids, coumarins, quinone, glycosides, steroids, saponins, carbohydrates, fatty acids, proteins, and amino acids were all identified through phytochemistry. A recent research has examined the health advantages of flavonoids, and their powerful antioxidant activities were demonstrated (Derakhshan et al., 2018) (Figure 1).

The aqueous extract from pomegranate peel possesses the highest concentration of phytochemicals, including phenols, terpenoids, glycosides, flavonoids, proteins, carbohydrates, and amino acids, of the four distinct extracts. The most efficient pomegranate peel extracts for preventing bacterial growth were those with acetone and ethanol (Karthikeyan & Vidya, 2019). One family of bioactive phytochemicals called phenolic compounds is mostly found in the pomegranate fruit's skin. Chlorogenic, syringic, caffeic, sinapic, ferulic, coumaric, vanillic, gallic, ellagic, and cinnamic acids are among the phenolic acids that have been found in pomegranate peel (PoP). In light of this, PoP should be viewed as a coproduce of juice extraction rather than as trash.

In order to extract the second metabolic product from the waste biomass, Banerjee et al. (2017) provided a thorough understanding of the techniques (such as liquid–liquid and solid–liquid extraction). The recovery of secondary metabolic products such as polyphenolic compounds has been greatly assisted by the use of alcohol recent extraction methods, sometimes called “green extractions”. These methods, which have produced higher levels of metabolite recovery than conventional extraction methods, include enzymatic and microwave‐assisted extractions. The typical extraction of metabolites begins with digestion, which leads to the breakdown of their structure and, later, to the recovery of polyphenols and pectins. It has been found that an extract containing a mixture of polyphenolic compounds performed better than the individual compounds (as a result of synergistic effects). The study by Akhtar et al. (2015) explained the effectiveness of pomegranate peel extract (PoPx) in extending the shelf life of a variety of food products. Although the usage of PoP waste is an intriguing area of research, unlike grape pomace, its extract has not yet been commercialized (Banerjee et al., 2017).

### 1.3 Effect of extraction techniques

Several factors, including the variety of fruits, the growth region, the agronomic climate, the farming practices, the processing factors, and storage conditions, have an impact on the secondary metabolites in pomegranate peel. These changes have an effect on the physical and chemical makeup of pomegranate fruit peel, which in turn has an effect on the quality of peel products. Al‐Rawahi et al. (2013) examined the impact of several solvents (such as methanol, ethanol, and water) used to extract phenolic compounds from peels of pomegranate. It was demonstrated that compared to pomegranate peel extracts in methanol and ethanol, the extract of peel in water had a greater capacity for extracting and a greater overall amount of phenols. The values of gallic acid equivalent(GAE)/100 g were 8460, 5990, and 4530 mg of dry‐meat equivalent (DM) for water, methanol, and ethanol, respectively. In order to extract essential oil constituents from pomegranate peel, supercritical fluid extraction was done. The outcomes were compared to those attained using the hydrodistillation method. While a quantity of 0.21% (v/w) was recorded for hydro distillation, the extraction of supercritical liquid yielded a higher extraction yield of 1.18% (v/w). An investigation by Magangana et al. (2020) revealed a high recovery of phenolic content. The researchers used the ability of various enzymes to macerate substances to create an efficient, rapid, and green extraction protocol for the ideal recovery of phenolic antioxidants from peel. It was verified that the recovery of crude extracts from supercritical liquid was doubled by enzymatic extraction, and it was reported that the total phenolic content increased to 301.53 mg of gallic acid equivalent (GAE) per g.

To maximize the production of phenolic compounds and the antioxidant activity of peel, Sharayei et al. (2019) examined and improved the technique of ultrasonically assisted extraction using water as the solvent. The study discovered that a 60% ultrasound amplitude and 6.2‐minute ultrasonic exposure time were the most optimal conditions. The maximum extraction and total phenolic contents of the pomegranate leaf extract were, respectively, 13.1% and 42.2 mg GA/g under these optimal conditions. The conditions for microwave‐assisted extraction of flavonoids from the peel were optimized, with the highest flavonoid production recorded being 4.26%. With success, the extract suppressed the radicals 2,2‐diphenyl‐1‐picrylhydrazyl.

## 2 HEALTH BENEFITS

Different parts of pomegranate fruit, such as peels, contain a large number of bioactive compounds that exhibit a variety of functions. In addition to inhibiting the oxidation of low‐density lipoproteins, peel extracts have significant antioxidant capacity in the removal of superoxide anion, peroxyl, and hydroxyl radicals. Kanatt et al. (2010) studied the antioxidant impact of pomegranate.

### 2.1 Antioxidant

Many ailments and diseases, including AIDS and cancer, are influenced by free radicals. Through its ability to scavenge free radicals, antioxidants assist in lowering the risk of disease and managing it. The use of natural antioxidants as substitutes for synthetic antioxidants that are less safe is growing steadily in the food industry (El‐Hadary & Taha, 2020; Mutahar et al., 2012). In this context, the literature has extensively discussed the antioxidant activity of PP extracts. The antioxidant strength of several extracts from the Pakistani pomegranate variety known as “Sufaid Alipuri” was assessed by Ismail et al. (2016). The extracts of acetone (427.2 mg GAE/g extract), methanol (367.9 mg GAE/g extract), ethanol (361.8 mg GAE/g extract), and water (273.5 mg GAE/g extract) had the highest total phenolic content (TPC). According to the analysis done using the FRAP method, the extracts of acetone also showed the highest level of antioxidant activity (91.4 mmol Fe/g), whereas the extracts of methanol had the highest level using the DPPH method. In a separate study, El‐Hadary and Ramadan (2019) found that the pomegranate methanolic extract had strong antioxidant activity, as measured using DPPH (93.97%) and ABTS (90.92%) methods, and had a total phenolic content (TPC) of about 189 mg GAE per g of extract.

### 2.2 Antimicrobial activity

It is a major issue everywhere in the world that many microbes are resistant to the available antibiotics. This has led researchers and the food industry to search for new alternative ingredients that may inhibit a wide range of microorganisms. In this vein, PP has been cited in numerous studies as a significant source of antimicrobial substances, which may protect food against microbial deterioration and reduce the incidence of dietary illnesses (Singh et al., 2018). According to Gullon et al. (2016), a 30–60 mg/mL methanol extract was effective against *Salmonella* sp., *Pseudomonas aeruginosa*, *Escherichia coli*, *Listeria monocytogenes*, and *Staphylococcus aureus*. An ethanolic extract of Tunisian PP, according to Wafa et al. (2017), was effective against a chicken meat‐isolated strain of *Salmonella Kentucky*. Minimum bactericidal concentrations (MBC) of 11.5 and 10.75 mg/mL, respectively, were obtained by the extract.

### 2.3 Antihypertensive

The angiotensin‐converting enzyme (ACE), which is thought to play a role in the renin‐angiotensin system's ability to regulate blood pressure, can be inhibited by phytochemicals found in PP. Arun et al. (2016) verified the effectiveness of an extract rich in polyphenols, primarily p‐coumaric acid, cinnamic acid, caffeic acid, and chlorogenic acid, as an ACE inhibitor. The presence of proteins and peptides was credited by Hernández‐Corroto et al. (2020) with contributing to PP's antihypertensive properties.

### 2.4 Anti‐inflammatory

With the help of punicalagin, ellagic acid, and punicalin, anti‐inflammatory effects are achieved. Aqueous PP extract at a dose of 10 g/mL reduced the secretion of CXCL8, a pro‐inflammatory cytokine with chemotactic activity toward T lymphocytes, basophils, and neutrophils, by 43% compared to the positive control, according to a recent study using an in vitro model of human colonic adenocarcinoma Caco‐2 cells (Mastrogiovanni et al., 2019). According to Stojanović et al. (2017), the phytochemicals present in the skin of the pomegranate may be crucial in the treatment of autoimmune conditions and chronic inflammation, including type 1 diabetes and multiple sclerosis. By stimulating macrophages, NK, dendritic as well as B and T cells, the PP‐separated pectins were able to modulate the inflammatory response of the immune system in a different study (Ahmadi Gavlighi et al., 2018).

### 2.5 Hepatoprotective effect

The liver is a major organ in the body that has multifunctional capabilities and plays a role, especially in the digestion of food, removal of toxins from the body, metabolism of drugs, acts as a storage site for numerous components such as glycogen, vitamins, and minerals, and regulates blood clotting time (Okaiyeto et al., 2018). Liver damage is a global health issue. Hepatic cells are damaged because of exposure to environmental toxins, misuse of over‐the‐counter and prescription medicines such as paracetamol (acetaminophen), isoniazid, fluconazole, etc., antineoplastic agents like carbon tetrachloride, viral and microbial infections, and excessive use of alcohol. Several phytochemical compounds have demonstrated effectiveness as hepatoprotective agents including Glycyrrhizin, Curcumin, Silymarin, Catechins, Quercetin, etc. (Okaiyeto et al., 2018). Several studies have demonstrated that pomegranate peel can help prevent hepatotoxicity and other liver problems. According to a study on mice with Concanavalin A‐induced hepatitis, the hepatoprotective benefits of pomegranate peel hydroalcoholic extract were mostly attributed to phenolic and flavonoid elements (Murtaza et al., 2021). A rise in liver enzymes such as alkaline phosphatase (ALP), aspartate transaminase (AST), and alanine transaminase (ALT), as well as oxidative stress indicators like serum total oxidant status (TOS) and malondialdehyde (MDA) levels, resulting in hepatic lesions and necrosis. Pomegranate peel extract and its bioactive ingredient Quercetin significantly reduced free radicals by decreasing serum oxidative stress markers TOS and MDA and increasing antioxidant enzyme activities (serum total antioxidant capacity [TAC], superoxide dismutase [SOD], catalase [CAT], and liver glutathione [GSH]) (Murtaza et al., 2021). In another study, it was demonstrated that intravenously administered pomegranate peel, prior to injecting thioacetamide, reduced liver injury by 60% (Al‐Sallami et al., 2018). Agrin, a peptidoglycan component of the membrane, is largely accumulated in liver cirrhosis and hepatocellular carcinoma. Expression of agrin was meager in rats treated with selenium 250 mg/kg and pomegranate peel 200 mg/kg before thioacetamide 200 mg/kg, but the agrin expression in rats treated with 400 mg/kg pomegranate peel was either scarce or completely diminished (Al‐Sallami et al., 2018). The overdose of paracetamol, one of the safest over‐the‐counter medicines, results in hepatotoxicity, which is generally treated with N‐acetyl cysteine. A comparison of the effect of N‐acetyl cysteine and aqueous extract of pomegranate peel on paracetamol‐induced hepatotoxicity revealed that when used in combination they can significantly decrease NO, MDA, ALT, AST, and total bilirubin and cause a marked increase in SOD, CAT, GSH, GPx, albumin, and total protein mean values (Abd El Fattah et al., 2018). The hepatoprotective properties are mainly assigned to pomegranate peel polysaccharides, which have super scavenging powers against free radicals (Zhai et al., 2018). Thus, pomegranate peel alone or in combination with other bioactive agents can be used to protect the liver against environmental toxins or side effects of xenobiotics and can reduce the risk of liver injury. However, more research is required to establish the safest therapeutic dose of bioactive ingredients in pomegranate peel for use in humans.

### 2.6 Neuroprotective effect

Neurodegenerative disorders are prevalent in the world owing to environmental stress, aging, genetics, and exposure to radiation. Worldwide, many people are affected by Parkinson's disease, Alzheimer's disease, stroke, epilepsy, migraines, and brain tumor. It is worth mentioning that polyphenols and other compounds in pomegranate and its bioactive waste have neuroprotective effects, as has been demonstrated by several studies. Pomegranate peel owes its neuroprotective effect majorly to proanthocyanidin compounds, ellagitannins, punicalagin, granatin, flavonoids, and phenolics compounds (Emami Kazemabad et al., 2022). In a neuronal environment, polyphenols extracted from pomegranate peel improve brain neurochemistry through their ability to inhibit NF‐κB actions, which is responsible for mRNA transcription of toxic and proinflammatory biomolecules. Pomegranate peel extracts also reduce cyclooxygenase 2 (Cox‐2) enzymatic activities and catalytic activities of the caspase enzyme. These bioeffects of PPE lead to the maintenance of neuronal hemostasis by reducing neuroinflammation and alleviating clinical manifestations of neuronal disorders/diseases (Mehdi et al., 2022). Adult stroke is the third leading cause of disability and the second leading cause of death internationally. It is a complex phenomenon that involves several pathological processes such as blood–brain barrier disruption, oxidative damage, apoptosis, excitotoxicity, and inflammation. Natural polyphenols' primary method of preventing stroke is their anticoagulant and antiplatelet properties (Parrella et al., 2021). Ellagic acid and ellagitannins prevented brain ischemia in both cellular and in vivo models by controlling the expression of Bcl‐2/Bax (Wang et al., 2019). Punicalagin, a natural ellagitannin found in pomegranates in high concentrations, reduced infarct volume and neurological deficits in rats given tMCAO through its antioxidant, anti‐inflammatory, and antiapoptotic properties (El‐Missiry et al., 2018; Parrella et al., 2021). The butanol and chloroform extract of pomegranate peel provided promising results against Alzheimer's disease, a slow‐progressing neurodegenerative disorder, by inhibiting acetylcholinesterase activity and exhibiting antioxidant action (Khokar et al., 2021). Pomegranate peel extract can lessen histopathological alterations in the brain brought on by electromagnetic radiation from mobile phones. Heat shock proteins are phosphorylated and activated by EMR, which increases oxidative stress and causes an increase in apoptosis. This inhibits the cytochrome‐caspase‐3 apoptotic pathway, which aids in the development of brain cancer. Flavonoids, polyphenols, and tannins in pomegranate peel provide a neuroprotective effect and decrease oxidative stress by inhibiting IL‐1β, TNF‐α, and NO which are activated in microglia cells (Belal et al., 2020).

### 2.7 Immunomodulatory activity

Pomegranate peel is a dietary polyphenol source that has been enhanced and has a powerful antioxidant activity (Ghasemi‐Sadabadi et al., 2021). It has effects against osteoporosis, hyperglycemia, diabetes, hypertension, neuroprotection, and immunomodulation (Fahmy & Farag, 2022). Dietary polyphenols may influence dendritic cells and have a significant immunomodulatory impact on macrophages, according to studies (Shakoor et al., 2021). The peel of a pomegranate also affects the growth of beta cells, and T cells, and suppresses Type 1, which also includes T‐cell helper cells such as Th1, Th2, Th17, and Th9 cells. In inflammatory bowel illness, polyphenols decrease inflammation by suppressing pro‐inflammatory cytokines, activating Treg cells in the gut, inhibiting tumor necrosis factor‐alpha (TNF‐α), and causing apoptosis, which reduces DNA damage. In auto‐immune illnesses such as type 1 diabetes, rheumatoid arthritis, and multiple sclerosis, it may have a role in both prevention and therapy by controlling signaling pathways, reducing inflammation, and restricting demyelination (Shakoor et al., 2021). Pomegranate peel intake activates B cells, which then develop into plasma cells and create Immunoglobulins (Ig A, IgG, IgM, and IgE), which function as antibodies to defend the immune system (Hachimura, 2018). Pomegranate peel extract may have an impact on the immunoregulatory‐related miRNA levels of adipose‐derived MSCs (Ad‐MSCs) since it has the capacity to reduce the expression of PI3KAKT1NF‐kβ genes implicated in inflammatory pathways through miRNA expression. In other words, peel extract could play a crucial part in controlling stem cells' immunomodulatory activity (Caruso et al., 2020).

### 2.8 Wound healing

Pomegranate peel extract (PPE), a polyphenolic substance obtained from pomegranates, has drawn a lot of attention for its anti‐inflammatory, bacteriostatic, and antioxidant properties. Pomegranate extract's ability to treat burn injuries was researched, and its probable processes were evaluated (Zhang, 2022). Due to the presence of ellagic, gallic, and other phenolic chemicals, pomegranate peel and seeds have wound‐healing capabilities (Hayouni et al., 2011). Additionally, pomegranate seeds contain high levels of pelargonidin‐3, 5‐diglucoside, cyanidin‐3, 3‐glucoside, pelargonidin‐3, 5‐glucoside, and delphinidin‐3‐glucoside (Hayouni et al., 2011). Coagulation and hemostasis, inflammation, proliferation, and wound remodeling are the main stages of the wound healing process. Collagen synthesis is stimulated by pomegranate consumption. The proliferation of fibroblastic cells, the primary source of collagen, is facilitated by the incredible growth factors generated by the macrophages, which is likely the cause of this impact (Bahadoram et al., 2022).

### 2.9 Anticancer activity

One of the major causes of death in the twenty‐first century is cancer. There has been an increase in interest in developing new potential therapies that could prevent, delay, or inhibit the growth of cancer in light of this disease's significant impact on human life and the costs to healthcare systems. PP extracts have the ability to inhibit the growth of tumor cells, according to certain researchers (Tortora et al., 2017). Gallic acid, ellagic acid, and punicalagin are the primary active ingredients in PP that have been linked to anticarcinogenic properties. Punicalagin and ellagic acid were also shown by Deng et al. (2017) to cause apoptosis in two human prostate cancer cell lines (DU145, PC_3_) and a mouse prostate cancer cell line (TRAMP‐C1). The findings of this study showed that the influence of PP on tumor cell death was mediated through an increase in the Bax/Bcl2 expression index and the activation of caspase‐3. Ellagic acid was also linked to anti‐proliferative effects on T_24_ cells from human bladder cancer. In a different study, Fazio et al. (2018) demonstrated that PP's isolated β‐glucan had anti‐proliferative properties against HeLa and MCF‐7 uterine cancer cells. The results of these studies generally imply that PP extracts could be used as an exciting new cancer treatment and prevention drug.

### 2.10 Antihyperglycemic activity

Diabetes is a metabolic disorder in which carbohydrate metabolism is disrupted due to either insufficient production of insulin or impaired sensitivity of cells toward insulin. Effects of a number of bioactive ingredients of ginger, garlic, cinnamon, ginseng, and glycyrrhiza glabra have been evaluated for their antihyperglycemic activity (Shi et al., 2019). Pomegranate peel projects antidiabetic activity by inhibition of α‐amylase and α‐glucosidases. Inhibition of these enzymes results in delayed digestion of carbohydrates, thus reducing the speed of glucose release in the blood (Ali Redha et al., 2018). By boosting insulin receptor protein and phosphorylated insulin receptor substrate 1, pomegranate peel polyphenols reduce insulin resistance. Additionally, these polyphenols activate the phosphoinositide 3‐kinase (PI3K)/protein kinase B (AKT/PKB) signaling pathway, the peroxisome proliferator‐activated receptor gamma (PPAR), and the glucose transporter 4 protein levels (GLUT4) (Zhang, 2022). Ellagic acid in pomegranate peel significantly decreased glucose fasting levels while increasing insulin sensitivity. Four‐week oral administration of 400 mg/kg/day of pomegranate peel alone or along with black bean peel extract ameliorated hyperglycemia (Amor et al., 2020). Pomegranate peel‐derived punicalagin interacts with α‐glucosidase, thus delaying its role in carbohydrate metabolism (Liu et al., 2022). Methanol extracts of pomegranate peel inhibit glycosylation of hemoglobin for exerting their antidiabetic effect (Sani & Nair, 2017). Studies have shown that pomegranate peel extract‐stabilized gold nanoparticles significantly decreased the risk of diabetes nephropathy by reducing glucose fasting levels, renal toxicity indices, and serum total cholesterol and triglycerides in a hyperglycemic condition. These nanoparticles are stable and have more bioavailability to mediate dephosphorylation of NF‐κB subunits, thus reducing the burden of proinflammatory cytokines in diabetic nephropathy (Manna et al., 2019) (Table 2).

**TABLE 2:** Review of physiological functions of *P. granatum L.*

Plant part	Bioactive compound	Physiological function	Details	References
Peel extracts	Punicalagin, punicalin, strictinin A, granatin B	Anti‐inflammatory	Pomegranate fractions substantially decreased carrageenan‐induced mice paw edema for 1, 3, and 4 h. They may also have prevented RAW 264.7 macrophages exposed to lipopolysaccharide (LPS) from releasing nitric oxide (NO)	Lee et al. (2010)
Peel extracts	Ellagitannins	Antioxidant	As an antioxidant substance, urolithin can lessen the renal oxidative injury brought on by cisplatin in kidney of mice. The levels of lipid peroxidation, oxidative biomarkers, TBARS, and Ox‐LDL associated with cardiovascular risk are decreased by pomegranate peel extracts in healthy ones	Jing et al. (2019)
Peel extracts	Phenolic and flavonoids compounds	Antihyperlipidemic, antihyperglycemic, and antioxidant properties	Due to their antioxidant components, peel extracts demonstrated antihyperglycemic and antihyperlipidemic properties. Moreover, the peel extracts improved the liver and kidney functions of diabetic and hyperlipidemic rats as compared to standard medications	El‐Hadary and Ramadan (2019)
Aril and peel extracts	Vitamin C	Antimicrobial (*E. coli*, *S. aureus*, and *P. aeruginosa*)	Fruit extracts had more antimicrobial activity than *P. aeruginosa* in comparison to *S. aureus*, whereas *E. coli* was resistant	Opara et al. (2008)
Pomegranate peels	Polyphenols, dietary fiber	Cardiovascular protection	Pomegranate peels include polyphenols and dietary fiber that lower total cholesterol, LDL, triglycerides, and lipid peroxidation levels, preventing cardiovascular disease	Hossin (2009)
Pomegranate peel extracts	Punicalagin, ellagic acid, ellagitannins	Anticancer	Pomegranate peel extracts have the ability to activate pro‐survival signal pathways and inhibit cell proliferation as well as the expression of angiogenic markers	Khan et al. (2009)
Pomegranate peel extracts	Ellagic acid, urolithins	Neuroprotection	Alzheimer's disease may be prevented or treated using elagic acid and its metabolite urolithin	Essa et al. (2015)

## 3 FOOD APPLICATIONS

Pomegranate is a wonder fruit and has numerous health benefits that have added to its existing popularity. Pomegranate juice, whether produced in industry or prepared at home, generates peel and seeds as residual by‐products. Quite a few studies have been done on this bioactive waste and it has been acknowledged that it has numerous valuable properties. Some of its utilization in food industries is given below.

### 3.1 Food additives, bio‐preservatives, and functional foods

#### 3.1.1 Milk and milk products

**FIGURE 2:**
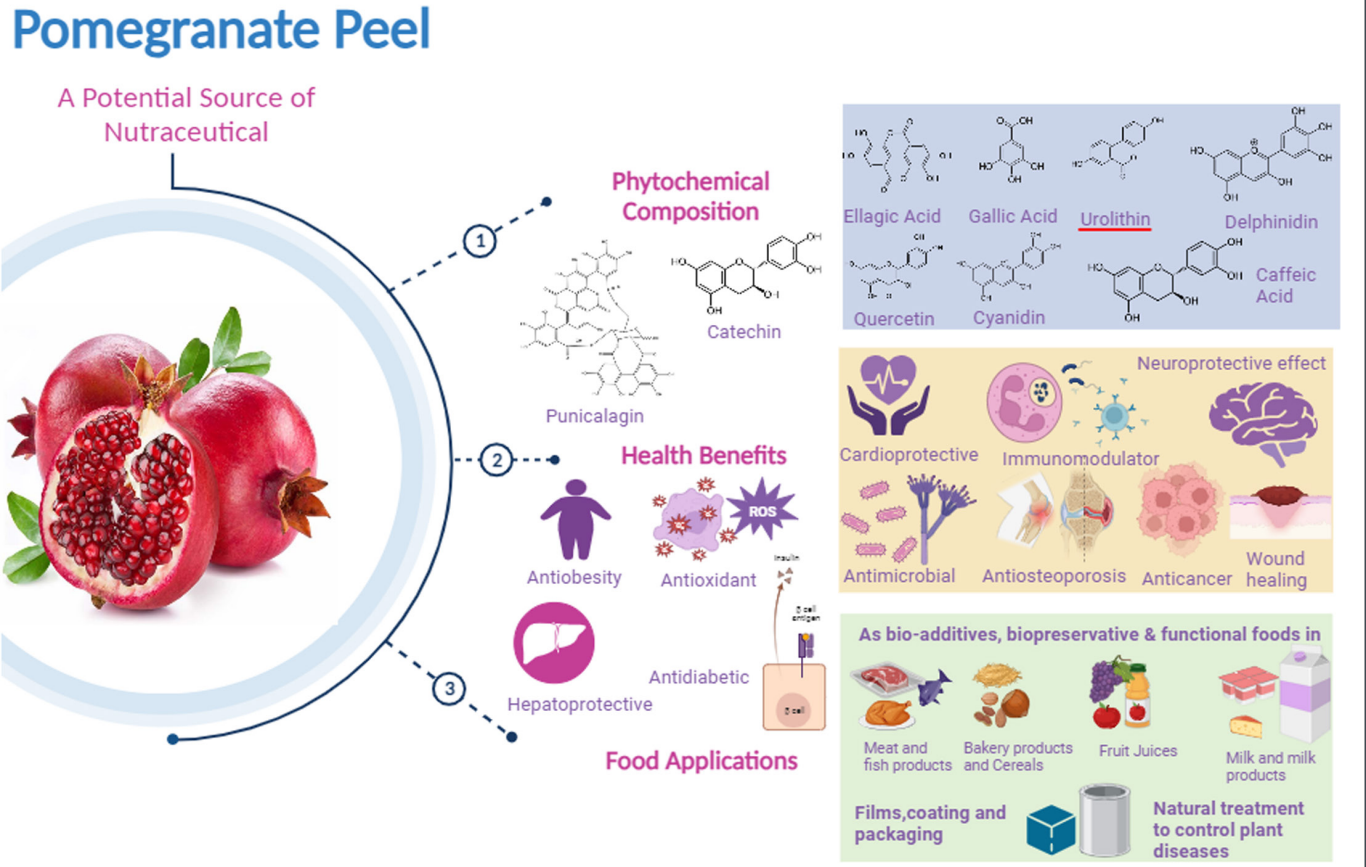
Pomegranate peel‐health benefits and food applications.

The polyphenols found in pomegranate peel extract have the advantages of being both natural antioxidants and bio‐preservatives. A study revealed that pomegranate peel extracts in concentrations of 150 and 300 mg/L, when added to fermented milk beverage along with *Lactobacillus* and *Bifidobacterium*, produced a high‐quality fermented probiotic beverage with increased antioxidant and antimicrobial activity (Al‐Hindi & Abd El Ghani, 2020). Pomegranate peel added to low‐fat bio‐yogurt improves both the probiotics' viability during storage as well as the final product's smoothness and viscosity (Ibrahim et al., 2020). Fortification of yogurt with honey and pomegranate peel by freeze drying yielded yogurt powder with higher polyphenolic content with better antioxidant properties and increased the glass transition temperature, increased the stability of the powder, and improved its shelf life (Kennas et al., 2020). Milk augmented with pomegranate peel aqueous extract has been used to produce cheese. The treated cheese, even with a low concentration of extract, showed more firmness and a lesser count of *Staphylococcus aureus* as compared to untreated cheese for up to 12 days of storage (Parafati et al., 2021). The use of organic by‐products as preservatives showed better results than the chemical food preservatives along with lesser side effects. Fortification of butter with pomegranate peel extract not only showed enhanced antioxidant properties but also low levels of free fatty acids and microbes, thus proving it to be a good bio‐preservative for butter (Ebrahimian et al., 2023). One of the additional benefits of using pomegranate peel extract in milk products is that in combination with probiotics, it effectively lowers the levels of triglycerides and reduces intracellular lipid accumulation (Figure 2).

#### 3.1.2 Meat, poultry, and fish

Due to increasing awareness regarding the relationship between health and diet, consumers are adamant about searching for food products with functional properties in addition to nutritional ones. Especially meat industry is actively searching for natural food preservatives in place of synthetic ones. This will protect the meat items' quality while also extending their shelf life (Gullón et al., 2020; Esfahani et al., 2022). Minced meat is one of the products that due to the oxidation of lipids and proteins loses its functional and nutritional qualities rather quickly and is more prone to spoilage due to pathogenic bacteria. However, pH, crude fiber, fat, ash, and protein content did not show significant (*p* > .05) fluctuations over time. The sensory evaluation revealed that chicken tender pops enriched with 6% PPP (T_2_) had high overall acceptability and balanced organoleptic properties. It can be concluded that PPP can be effectively used as a natural source of fiber, antioxidant, and antimicrobial agent in novel functional foods (Basharat et al., 2023). Pomegranate peel, due to polyphenolic and flavonoids, not only effectively decreased lipid oxidation and confirmed its robust natural preservative action for minced beef preservation, but also showed antibacterial activity against food‐borne pathogens such as *S. enterica*, *P. aeruginosa*, and *E. coli* (Fourati et al., 2020). The use of pomegranate peel and its extracts in meat products would significantly minimize the requirement for synthetic antioxidants like butylated hydroxytoluene (BHT), tert‐butylhydroquinone (TBHQ), and others that the food industry avoids due to poor toxicological reports (Smaoui et al., 2019).

Dietary supplementation of boiler chicken (kept under heat stress) with pomegranate peel extracts up to 650 mg/kg not only reduced triglycerides, low‐density lipoproteins, alkaline phosphatase, and alanine aminotransferase, but also increased the high‐density lipoproteins and weight of the chicken with better intestinal morphology. In addition to this, supplementation with pomegranate peel extract also preserved meat quality during refrigeration (Sharifian et al., 2019). One study conducted to test the decontamination potential of pomegranate and grape waste extract on poultry carcass in a slaughterhouse proved that these natural antimicrobial agents kept the treated chicken decontaminated and increased its shelf life up till 9th day of storage (Dakheli, 2020). Coating of chicken nuggets with pomegranate peel powder and sodium alginate increased the nutritional value, quality, and safety by decreasing the total aerobic microbial count and increasing the oxidative stability of the product up till the 21st day of storage (Bashir et al., 2022).

In a recent study, it was proved that both aqueous and ethanolic pomegranate peel extract decreased the deterioration of bighead carp fillets and increased the shelf life of the product by maintaining the appearance, quality, and stability of the product and preventing microbial flora count during cold storage (Zhuang et al., 2019). The addition of pomegranate peel extract to the coating of ready‐to‐cook breaded cod sticks increased their nutritional quality, delayed microbial growth, and prolonged their shelf life during refrigeration (Panza et al., 2021).

#### 3.1.3 Baked goods and cereal products

Comparison of supplementation of two different soymeal diets (corn vs. rice‐sorghum) with pomegranate peel concluded that rats fed with pomegranate peel supplemented soymeal diet have serum cholesterol levels, triglycerides, and plasma glucose levels were reduced irrespective of cereal source (Gopi et al., 2019). Making muffin cakes with wheat flour substituted with pomegranate peel powder showed that the extract enhanced the viscosity of the batter and increased the amount of total dietary fiber, phenolic content, and mineral content in the muffins. Additionally, the antioxidant activity was raised, and the muffins' flavor, aroma, general texture, and nutritional value were improved (Topkaya & Isik, 2019). The protein, fat, and ash content of the biscuits were unaffected by the addition of pomegranate peel, but the antioxidant activity, total phenolic content, and dietary fiber content were all raised. However, it was concluded that powder substitution should not be increased by more than 12% as it will contribute to the enhanced sour and bitter taste of the biscuits making them inedible (Urganci & Fatma, 2021). Date bars containing date paste, nuts, and roasted gram, when fortified with pomegranate and apple peels, showed that both can be used as therapeutic agents against degenerative disorders due to oxidative stress due to their high polyphenolic content and associated antioxidant activity (Ranjha et al., 2020).

### 3.2 Film, coating, and packaging

In recent years, there has been a rise in popularity for the use of fruit waste to create films, recycling materials, and wrapping materials. The antioxidant properties of films and recycling materials may be improved by PPx due to its high phenolic content. The main ellagitannin in the fruit, punicalagin, is a phenolic compound found in the pomegranate peel that is responsible for the fruit's antioxidant activity. When used to create starch‐based films, pomegranate peel functions as an antibacterial and a strengthening agent. A study showed that pomegranate peel's antibacterial action prevented the development of both gram‐positive and gram‐negative bacteria, and because the peel's semi‐crystalline structure was preserved, it also boosted the tensile strength and stiffness of starch‐based films (Ali et al., 2019). Pomegranate peel powder was added to chitosan films to produce food‐grade packaging materials that had improved antioxidant and antibacterial properties as well as increased film thickness, water permeability and solubility, and total phenolic content with reduced humidity and mechanical characteristics (Zeng et al., 2021).

It has been proven that edible caps made of chitosan and pomegranate peel extract can stop food from becoming contaminated microbiologically. This mixture reduced the overall number of aerobic plaques (the total amount of bacteria), and it was observed that the pH and total volatile organic nitrogen (TVB‐N) levels in the Pacific Ocean's white camarones were inhibited. Additionally, the rise in chemical decomposition measurements such as TVB‐N and TBARS for Nile tilapia fillets was observed (Alsaggaf et al., 2017).

### 3.3 Natural treatment to control plant diseases

Synthetic pesticides are widely used, which have negative effects on the environment and human health. According to investigations, using pomegranate peel byproducts will offer an inventive and environmentally friendly way to stop plant diseases and food deterioration. Due to its high efficacy and broad‐spectrum action against bacteria, fungi, and viral pathogens, it is a suitable alternative to artificial pesticides (Belgacem et al., 2021). Due to its antifungal properties, it can be used to control green and blue mold post‐harvest invasion of citrus fruits. Pomegranate peel extracts were shown to be effective against *Penicillium digitatum*, and recommendations were made to disinfect storage rooms and recirculated water in packing houses using pomegranate peel extract sanitizers for total control of green mold in citrus fruits (Pangallo et al., 2021). In another study, spraying pomegranate peel extract at 12 g/L on olive plants reduced the risk of olive anthracnose by 84.5% and prevented premature defoliation of plants (Pangallo et al., 2022).

## 4 CONCLUSION AND FUTURE DIRECTIONS

Pomegranate peel and its extract have a lot of potential as a valuable source of nutraceuticals, as shown by their phytochemical profile and nutritional makeup. Numerous health advantages, including antioxidant, anti‐inflammatory, anticancer, and cardiovascular protective characteristics, have been linked to the wide variety of bioactive chemicals contained in pomegranate peel, including polyphenols, flavonoids, tannins, and anthocyanins. Pomegranate peel is notable for its nutritional makeup, which includes high concentrations of dietary fiber, vitamins (especially vitamin C), and minerals like potassium. Pomegranate peel and extract are a desirable alternative for inclusion in functional foods and supplements because of their components, which add to their overall nutritional worth. Additionally, studies have demonstrated that pomegranate peel extract has therapeutic promise for treating a range of illnesses, including diabetes. Due to its potential uses in the creation of nutraceutical goods, the extraction of bioactive components from pomegranate peel has attracted a lot of interest. With its high phytochemical content, pomegranate peel extract can be used to harness the health‐promoting qualities of dietary supplements, functional meals, and beverages. While pomegranate peel and peel extract show tremendous promise as sources for nutraceuticals, further study is necessary to fully understand their mechanisms of action, bioavailability, and potential negative effects in different populations. To guarantee the security and effectiveness of food products made from pomegranate peel, concerns regarding sourcing, processing, and quality control should also be addressed. In conclusion, pomegranate peel and peel extract have become promising bio‐wastes in the field of nutraceuticals, providing a pure and powerful source of bioactive chemicals that can improve health and prevent disease. The discovery of novel nutraceutical items that can improve human well‐being is anticipated to result from further investigation of their therapeutic potential and ongoing research activities.

## AUTHOR CONTRIBUTIONS

**Faiza Azmat:** Writing – original draft (equal). **Mahpara Safdar:** Visualization (equal). **Hajra Ahmad:** Validation (equal). **Muhammad Rizwan Jamil Khan:** Software (equal). **Junaid Abid:** Data curation (equal). **Muhammad Sadiq Naseer:** Writing – review and editing (equal). **Saurabh Aggarwal:** Formal analysis (equal). **Ali Imran:** Supervision (equal). **Urma Khalid:** Writing – original draft (equal). **Syeda Mahvish Zahra:** Supervision (equal); writing – review and editing (equal). **Fakhar Islam:** Supervision (equal). **Sadia arif cheema:** Data curation (equal). **Umber Shehzadi:** Visualization (equal); writing – review and editing (equal). **Rehman Ali:** Writing – review and editing (equal). **Abdela Befa Kinki:** Software (equal). **Yuosra Amer Ali:** Validation (equal). **Hafiz Ansar Rasul Suleria:** Visualization (equal); writing – review and editing (equal).

## CONFLICT OF INTEREST STATEMENT

The authors declare that they have no conflict of interest.

## ETHICAL STATEMENT

This article does not contain any studies with human participants or animals performed by any of the authors.

## CONSENT TO PARTICIPATE

Corresponding and all the co‐authors are willing to participate in this manuscript.

## CONSENT FOR PUBLICATION

All authors are willing for publication of this manuscript.

## Data Availability

Even though adequate data has been given in the form of tables and figures, however, all authors declare that if more data required, then the data will be provided on request basis.
